# Study of Physiological Adaptations in Vertical Kilometer Runners: Focus on Cardiorespiratory and Local Muscle Demands

**DOI:** 10.3390/jfmk9040230

**Published:** 2024-11-12

**Authors:** Pablo Jesús Bascuas, Héctor Gutiérrez, Eduardo Piedrafita, Ana Vanessa Bataller-Cervero, César Berzosa

**Affiliations:** Facultad de Ciencias de la Salud, Universidad San Jorge, Autov. A-23 Zaragoza-Huesca, KM 299, 50830 Villanueva de Gállego, Zaragoza, Spain; pbascuas@usj.es (P.J.B.); hgutierrez@usj.es (H.G.); epiedrafita@usj.es (E.P.); cberzosa@usj.es (C.B.)

**Keywords:** field tests, physiological assessment, sport performance, portable devices, trail running

## Abstract

**Background:** Research into key performance factors in trail running, particularly in vertical kilometer (VK) races, is crucial for effective training and periodization. However, recent studies on metabolic and cardiorespiratory responses during VK races, especially using field tests, are limited. **Objectives:** Therefore, the aim of this study is to evaluate the metabolic and cardiorespiratory responses during a VK field test, identifying differences based on sex and performance level, as well as key performance factors and their deterioration due to fatigue. Fifteen trained trail runners (ten males and five females, 19 to 38 years old) perform a VK race. **Methods:** The global physiological response is evaluated using the portable gas analyzer Cosmed K5 and the local response using near-infrared spectroscopy technology. **Results**: In gender comparisons, the ANCOVA test shows significant differences (*p* < 0.05) in the ventilation, tidal volume, expiratory time-to-inspiratory time ratio, inspiratory flow rate, end-tidal CO_2_ partial pressure, heart rate, oxygen pulse, and total hemoglobin. Additionally, the performance comparison reveals significant differences in the variables’ velocity, oxygen consumption, carbon dioxide production, ventilation, dead space-to-tidal volume ratio, total time of the breathing cycle, expiratory time-to-inspiratory time ratio, inspiratory duty cycle, expiratory fractions of CO_2_, quadriceps saturation index, and VE/VCO_2_ ratio. Finally, the correlation analysis shows oxygen consumption (r = −0.80 mean; r = −0.72 peak), carbon dioxide production (r = −0.91 mean; r = −0.75 peak), expiratory time-to-inspiratory time ratio (r = 0.68 peak), ventilation (r = −0.58 mean), and quadriceps saturation index (r = 0.54 mean; r = −0.76 coefficient of variation) as the key performance factors in the VK race. **Conclusions:** Overall, the physiological analysis indicates the importance of local muscular adaptations and respiratory system capacity in this type of short-duration race.

## 1. Introduction

In recent years, an increase in the number of athletes participating in outdoor sports, such as trail running, has been observed. Within this sport, a wide variety of races with different durations can be identified. The investigation for key parameters in running performance, both road and trail, has progressively become a study aim for numerous researchers in the health and sports sciences field with regard to applying this knowledge to the formulation of training programs and periodization schedules for runners.

Recent studies have identified multiple physiological and biomechanical variables correlated with trail running performance, including maximal aerobic speed (VO_2_max), sustained fraction of VO_2_max, time limit of maximal aerobic speed, running economy, knee extensor maximal isometric voluntary force, and its decline or sustainable running power [1,2]. These variables are directly involved in maintaining increased running paces throughout the entire race, so their improvement could minimize the substantial acid–base imbalance that may compromise muscle contraction [2]. Additionally, it has been observed that these variables are related to the delay in muscle glycogen depletion during exercise, thus prolonging fatigue onset [3]. Furthermore, these variables are associated with an enhanced impact attenuation during the braking phase in downhill running, facilitated by the eccentric control on knee flexion, while in the propulsion phase, they contribute significantly to energy generation during running on steep slopes [1].

Due to the complexity of performance analysis in trail running races, recent studies have conducted investigations isolating uphill and downhill running to obtain more applicable conclusions. The two main performance predictors in both uphill and downhill running, in order of importance, are maximal aerobic speed and maximum strength, which could help maintain a better stride frequency and length. Nevertheless, there are third and fourth predictors, which are less decisive but relevant, that differ depending on the inclination type: body mass index (BMI) in uphill running and leg stiffness in downhill running [4]. Muscle tone and stiffness remain constant throughout the race after the initial adjustment of running kinematics, regardless of speed and inclination [5], while BMI has shown an inverse correlation with running economy [6].

Amongst the trail running modalities, the most popular races are characterized by a short duration and high intensity, such as the vertical kilometer, where runners are required to overcome an approximately 1000 m vertical elevation along a 5000 m distance. Regarding the previously mentioned physiological and biomechanical parameters, except for maximal aerobic speed, these have limited predictive values for short-trail running performance [7]. In fact, it is more appropriate to consider other variables, such as muscle endurance (lower fatigue index in the concentric knee extension torque, which could restrict muscle recruitment and coordination pattern changes), running economy when at a 10% incline, and lipid metabolism at a 10 km/h speed, as performance factors for these running distances [7]. Likewise, analyzing specifically the vertical kilometer race performance, the limited scientific evidence has highlighted the importance of maintaining elevated levels of vertical mechanical power, metabolic power (oxygen consumption and carbon dioxide production interaction), and vertical velocity throughout the entire race duration, regardless of the slope [8].

Regarding performance differences based on sex, it is well known that men tend to outperform women by approximately 10–30%, depending on the strength demands of the sport event. The largest differences are found in short-duration events that require significant amounts of strength and power. Men, due to higher testosterone secretion from adolescence, have greater muscle mass and strength, providing them with a competitive advantage in such short events [9,10]. However, this difference appears to decrease to about 1–3% in ultra-endurance events [11]. Despite this, there is still no scientific literature investigating sex-based performance differences in short-trail running events, such as the vertical kilometer.

Technological advancements have developed novel noninvasive portable devices that enable the real-time assessment of cardiorespiratory responses and hemodynamic skeletal muscle behavior during exercise. These devices facilitate the monitoring of the heart, respiratory system, and muscular physiological adaptability to changes in exercise intensity. Moreover, analyzing the ventilatory response to exercise, the ability of the trail runner to maintain metabolic power during the race could be assessed. This metabolic power, as a performance factor [8], is dependent on the VO_2_ and VCO_2_ by reflecting the amount of oxygen consumed by the muscle, which could serve as an indicator of skeletal muscle oxidative capacity during exercise [12]. They also allow for the measurement of local oxygenation responses (delivery vs. consumption) in the skeletal muscle microcirculation, providing real-time data on changes in oxyhemoglobin, deoxyhemoglobin, total hemoglobin, and the tissue saturation index [13]. The evaluation of skeletal muscle oxygenation using near-infrared spectroscopy (NIRS) depends on several key factors such as blood flow, hematocrit levels, muscle capillarity, muscle tissue metabolic state, mitochondrial density, oxidative capacity, and alterations in hemoglobin dissociation curves [14]. This technology enables the analysis of the arteriovenous oxygen difference in athletes, which plays a critical role in their oxidative capacity and endurance performance [15]. It serves as an indicator of oxygen delivery and consumption in skeletal muscle tissue, which are dependent on multiple factors such as enzymatic activity, mitochondrial density, and capillary density [15]. The number of recent publications in the endurance training field where NIRS technique is used has undergone an exponential increase in last years. These studies have revealed that NIRS is useful for stablishing training intensity zones based on the threshold breakpoints determination [13]. Furthermore, NIRS is able to determine the performance in short-length endurance events, being a reliable indicator of critical velocity and critical power, and may predict the exhaustion time during severe full-body exercise (like cycling and running above critical power) [13]. In particular, the application of NIRS in trail running allows for more sensitive control of exercise intensity compared to traditional parameters such as the heart rate [16]. Moreover, NIRS is less affected by changes in temperature, humidity, hydration, emotional state, altitude, and day hour [12]. Nevertheless, despite the mentioned advantages and portability, there are still few studies that use this technology in the trail running context. Considering the current publications, the majority of vertical kilometer trail running research has been conducted on a lab treadmill, while a few have been carried out via field tests. Consequently, the purpose of this study is to analyze metabolic and cardiorespiratory responses, both global and local, during a vertical kilometer field test using a portable gas analyzer and NIRS technology. The results of such physiological analysis in field tests could reveal crucial data for their application in training and for preparing these particular types of tests.

The primary objective is to observe the full-body metabolic and cardiorespiratory responses during an extremely short endurance-trail running event, aiming to identify differences based on performance level and sex, as well as to infer diverse performance factors in a real vertical kilometer field test. As a secondary objective, we aim to examine the impact of these performance factors on fatigue in this running modality.

We hypothesized that physiological adaptation would differ based on sex and the training level of the subjects, with men and more highly trained runners achieving superior values in cardiovascular, respiratory, and local muscular parameters during the test.

## 2. Materials and Methods

### 2.1. Experimental Design

**Figure 1:**
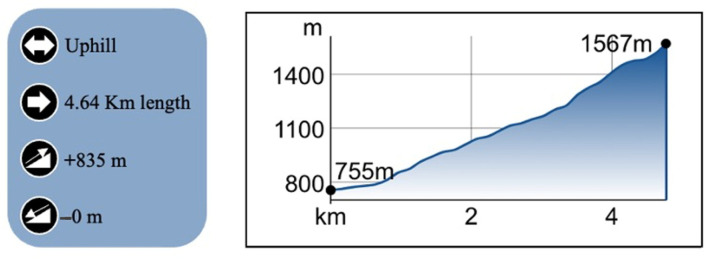
Vertical kilometer track.

This cross-sectional study involved each runner performing a vertical kilometer field test. A vertical kilometer is characterized by a maximum distance of 5 km with a positive elevation gain of 1000 m. In our case, the selected field test was 20% shorter than an official vertical kilometer, consisting of a continuous ascent over a distance of 4.64 km, with a positive elevation gain of 835 m (Figure 1). Despite the differences from the official race, the physiological response to continuous uphill running is expected to be very similar.

The itinerary included a combination of trail segments and forest track sections, with a moderate level of technical difficulty. The test was conducted under the most standardized conditions possible, aiming for a consistent temperature between 20 and 30 degrees Celsius. Due to the restriction on eating or drinking during the test, which was imposed using the gas analyzer, all participants were instructed to consume carbohydrates during the meal prior to the test to ensure full glycogen stores, as well as to maintain a good level of hydration in anticipation of dehydration during the test. Furthermore, no participant was allowed to consume caffeine or other supplements with ergogenic effects prior to the test.

### 2.2. Participants

Fifteen trained trail runners participated in this study (ten males and five females). The inclusion criteria required that each participant had been training regularly in trail running for more than 3 years, was accustomed to training and competing in vertical kilometer events, agreed to participate voluntarily, and signed an informed consent form. The exclusion criteria included having experienced any musculoskeletal injuries within the past year, currently having a febrile illness or infection, or presenting any pathological condition at the time of the study. The runners were recruited through contact with coaches from the Aragón Trail Running Federation. Prior to the experiment, all subjects were informed about the objectives, benefits, and risks of the research. All of them signed an informed consent form. The experimental protocol was approved by the University Ethics Committee (Ref. 005-19/20). All procedures fulfilled the Declaration of Helsinki requirements.

### 2.3. Measurements

#### 2.3.1. Portable Gas Analyzer Data

The participants were equipped with a portable gas analyzer (Cosmed K5, Rome, Italy) during the entire route to collect central metabolic and cardiorespiratory data breath by breath with a turbine flowmeter connected to an adjustable face mask. The gas analyzer was attached to the runner’s back with a harness, with the weight of the entire system being 900 g. All data were collected in the device recorder for subsequent analysis. The calibration process of the K5 system was performed before each test with a 3 L calibration syringe for the turbine. O_2_ and CO_2_ sensors were calibrated to the ambient air conditions (20.93% O_2_; 0.03% CO_2_), along with delay calibration, according to the manufacturer’s instructions. Before the VK test, the metabolic rate was determined during a 10 min standing trial. During the entire VK track, the following variables were assessed: velocity; VO_2_; VCO_2_; minute ventilation (VE); respiratory rate (RR); tidal volume (TV); dead space-to-tidal volume ratio (VD/TV); inspiration time (IT); expiration time (ET); total time of respiratory cycle (TotalT); expiratory time-to-inspiratory time ratio (ET/IT); relationship between inspiratory time and the total time of respiratory cycle, or inspiratory duty cycle (IT/TotalT); relationship between tidal volume and inspiratory time, or inspiratory flow rate (TV/IT); expiratory fractions of O_2_ (FEO_2_); expiratory fractions of CO_2_ (FECO_2_); fraction of inspired oxygen (FIO_2_), end-tidal O_2_ partial pressure (PETO_2_); end-tidal CO_2_ partial pressure (PETCO_2_); respiratory exchange ratio (RER); heart rate (HR); relationship between oxygen uptake and heart rate, or oxygen pulse (VO_2_/HR); VE/VO_2_ ratio; and VE/VCO_2_ ratio. The portable gas analyzer system was also equipped with a strap to monitor the HR and a GPS sensor to track the position and speed.

The mean values and those associated with maximum intensity effort (whether peak or minimum, depending on the analyzed variable) of all the parameters recorded during the VK test were evaluated. The variability of all variables was assessed using the coefficient of variation (CV) calculation in order to be able to analyze the ability to adapt their physiological response to changes in the terrain incline in order to maintain the fastest pacing possible within each runner’s personal capabilities.

#### 2.3.2. Near-Infrared Spectroscopy (NIRS) Data

A portable NIRS device (Moxy Monitor, Fortiori Design LLC, Hutchinson, MN, USA) was positioned on each runner’s right vastus lateralis (12 cm above the patellar proximal border) and 3–5 cm laterally to the thigh midline to monitor the peripheral tissue oxygenation. This measurement was used to standardize the placement of the device at the center of the vastus lateralis muscle belly of the quadriceps in all subjects. The NIRS device was strapped and attached with adhesive tape to secure the placement throughout the VK test. Near-infrared light is emitted by a diode at different wavelengths ranging from 630 to 850 nm into the skeletal muscle tissue. Hemoglobin (Hb) and myoglobin (Mb) absorb and reflect some of the emitted light at specific wavelengths and in different ways when they are oxygenated and deoxygenated [17]. The reflected near-infrared light is collected by two optical detectors positioned at 12.5 mm and 25.0 mm from the emitters. With this technology, oxyhemoglobin (OHb), deoxyhemoglobin (HHb), and total hemoglobin (THb) were assessed in the skeletal muscle representing the local oxygen delivery and consumption, along with alterations in the tissue blood volume. From these parameters, the muscle oxygen saturation index (SmO_2_), expressed as percentage, can be calculated using the following Equation (1):

(1) $${SmO}_{2}=\frac{oxyhemoglobin+oxymioglobin}{total\;hemoglobin+total\;mioglobin}\times 100$$

Hb and Mb cannot be distinguished using this technology, so they were assessed simultaneously.

### 2.4. Statistical Analysis

Student’s parametric *t*-test was conducted to detect significant differences between performance levels for both cardiorespiratory and metabolic parameters. For comparisons between different performance levels, the sample (including both men and women) was initially divided into quartiles based on the VK final time, using SPSS software version 28.0 to define these groups. Specifically, the VK final times were ranked into four quartiles using the “Rank Cases” function in SPSS, which assigns each runner a quartile based on their performance. This process resulted in 5 runners in the 1st quartile (the fastest) and 10 runners distributed across the 2nd, 3rd, and 4th quartiles (the slower runners), with runners in the 1st quartile completing the VK test in under 47.4 min. This division enabled us to focus our analysis on comparing the top-performing runners with those at lower performance levels, aligning with our aim to understand key differences in physiological adaptations. This approach also reflects previous findings in endurance racing, where higher-level runners—especially in shorter races—demonstrate lower pacing variability and higher velocities, which is relevant to the context of our study [18]. 

The physiological responses between first quartile runners were analyzed in comparison to the remaining quartiles runners. Analysis of covariance (ANCOVA) was conducted to detect significant differences between gender as a dependent variable and performance level (VK final time) as the covariate. The analysis of statistical power and effect size using Hedges’ g and gANCOVA were also conducted; gANCOVA was calculated using the following Equation (2):

(2) $$gANCOVA=g\times \sqrt{1-R_{ANCOVA\;adjusted}^{2}}$$

The effect size was categorized as small (d = 0.2), medium (d = 0.4), or large (d ≥ 0.8). Pearson’s correlation coefficient was calculated to identify potential variables correlated to the VK final time. A significance level of *p* < 0.05 was established. In the result tables of this study, statistical power calculations have been included to account for type II errors. Changes are considered sufficiently robust only if they have a statistical power value of 1-β greater than 0.80. However, the sample size used in this study is limited to discussing only very large effect sizes for Hedges’ g (greater than 1.5). To achieve a power analysis for large effect sizes (g = 0.8), a minimum sample size of 55 participants would be necessary. All these tests were conducted using the statistical package SPSS version 28.0 (SPSS, Chicago, IL, USA).

## 3. Results

Anthropometric data and training characteristics of the subjects are presented in Table 1.

The ANCOVA test with sex as the dependent variable and performance level as the covariate revealed significant differences in the results, as presented in Table 2.

**Figure 2:**
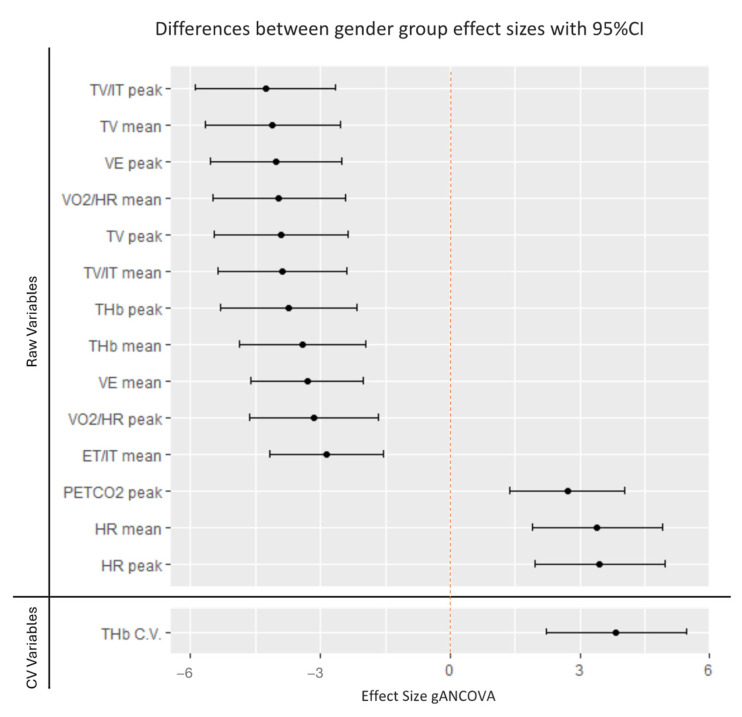
Gender group effect sizes. Effect sizes gANCOVA are calculated for each dependent variable. The graph represents the magnitude of the difference between sexes. Black lines with dots represent gender group effect size differences with 95% confidence intervals (CIs).

The analysis showed significant differences (*p* < 0.05) in the VE, TV, ET/IT, TV/IT, PETCO_2_, HR, VO_2_/HR, and THb. The effect size analysis is presented in Table 2 and Figure 2.

Student’s *t*-test results between performance levels are presented in Table 3. The runners’ distribution between the first quartile and the remaining quartiles to distinguish performance levels was calculated based on their VK field test final time. Finally, the runners in the first quartile finished in a final time below 47.4 min, and those who exceeded this threshold were placed in the rest of the quartiles.

**Figure 3:**
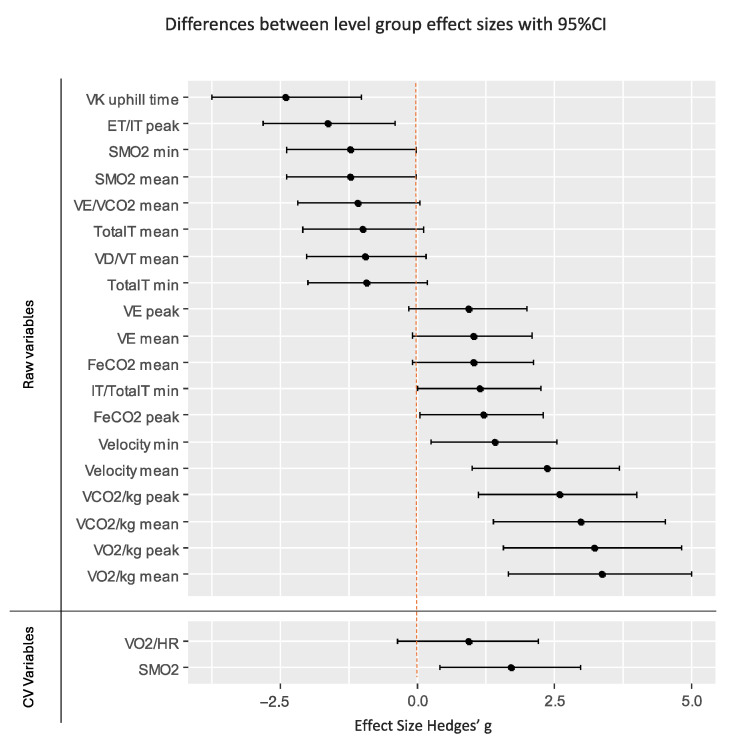
Level group effect sizes. Effect size Hedges’g for each dependent variable. The graph represents the magnitude of the difference between performance levels (1st quartile runners versus remaining quartiles). Black lines with dots represent level group effect size differences with 95% confidence intervals (CIs).

The inferential analysis revealed significant differences (*p* < 0.05) in the following variables: velocity, VO_2_, VCO_2_, VE, VD/TV, TotalT, ET/IT, IT/TotalT, FECO_2_, SmO_2_, and VE/VCO_2_. Moreover, in the variability analysis, the coefficient of variation for the variables VO_2_/HR and SmO_2_ exhibited significant differences based on the performance level. The effect size analysis, as presented in Table 3 and Figure 3, shows that all the significant changes revealed large effect sizes (≥0.8).

**Figure 4:**
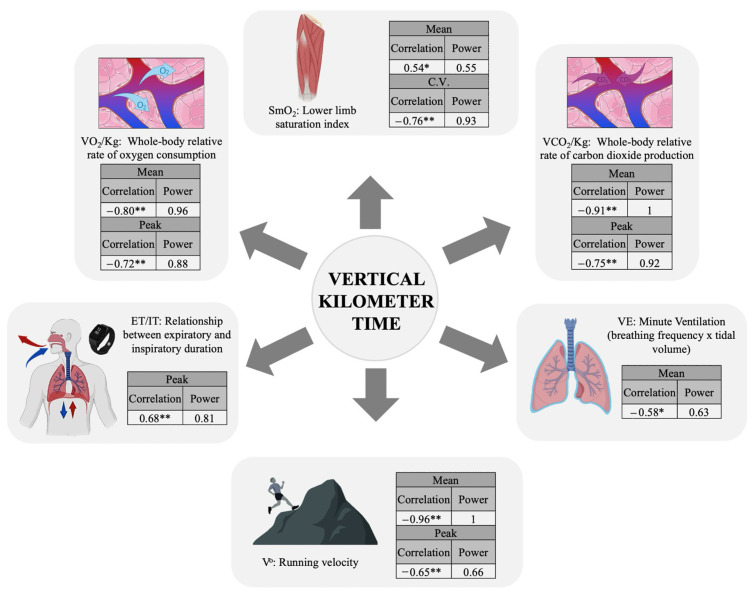
Vertical kilometer performance factors. * *p*-value < 0.05; ** *p*-value < 0.001.

The results of Pearson’s correlation analysis are illustrated in Figure 4. The findings identify the variables VO_2_, VCO_2_, velocity, SmO_2_, ET/IT, and VE as key performance factors in the VK field test. Positive correlations were observed between the uphill time and SmO_2_ and ET/IT, while negative correlations were observed for the remaining variables. All these correlations were statistically significant.

## 4. Discussion

The physiological demands in trail running have been a focal point of research in recent years, with a recent emphasis on short-duration races assessments and the simulation of vertical kilometer gradients through treadmill tests. However, to the best of our knowledge, there have been no physiological studies during the execution of a vertical kilometer field test simulating real-race conditions. Therefore, the objectives of this study were to evaluate the physiological demands, aiming to observe disparities based on sex and performance level, while concurrently identifying key parameters correlated with the final VK duration and their connection to the onset of fatigue.

### 4.1. Sex-Related Differences

The analysis of sex differences at equivalent performance levels has revealed statistically significant differences in the variables VE, TV, ET/IT, TV/IT, PetCO_2_, HR, oxygen pulse, and THb.

Concerning the respiratory system, the observed lower values of VE, TV, and TV/IT in women disappear when adjusting for the runner’s weight, suggesting that the diminished pulmonary capacity in women may underline these differences. These findings align with prior research highlighting anatomical differences in women, including a lower lung volume, smaller rib cage size, shorter diaphragm, greater contribution of inspiratory rib cage muscles, lower pulmonary volumes and flows, lower airway diameters, and smaller diffusion surface compared to men [19,20].

Moreover, these differences may contribute to limitations in expiratory flow, increased inspiratory resistive work, the work of breathing, and utilization of ventilatory reserves [19,20]. Despite the absence of elastic differences in the lungs and thorax between sexes, the lower pulmonary capacities and higher inspiratory and expiratory resistive work of breathing in women could account for the lower inspiratory flow rate (TV/IT) [21]. This variable is linked to central inspiratory drive and tachypneic breathing patterns and is considered the main factor conditioning the stability of ventilatory efficiency [22]. Concerning the central regulation of breathing patterns, the persistent lower expiration-to-inspiration time ratio (ET/IT) in women, even after weight normalization, indicates that women exhibited shorter expiratory durations and longer inspiratory durations during exercise. This outcome is associated with higher respiratory rates at a given ventilation during high-intensity exercises, resulting in an augmented energy cost of breathing and anticipatory respiratory muscle fatigue [23,24].

The higher mechanical restrictions on tidal volume and minute ventilation observed in women may necessitate a greater effort to maintain gas exchanges [19] and predispose them to develop expiratory flow limitation (inability to further increase expiratory flow despite increasing expiratory muscle effort) during high-intensity exercise [25]. These findings suggest that women may need to distribute a greater fraction of whole-body oxygen consumption and cardiac output to respiratory muscles, potentially competing with lower-body skeletal muscle blood flow during heavy exercise. The absence of observed blood flow redirection may be attributed to a lower sympathetic activity increment during exercise compared to males [19]. The compromised gas exchange may additionally result in lower alveolar ventilation, leading to exercise-induced arterial hypoxemia and an increase in arterial carbon dioxide [26], and, therefore, potentially explain the higher end-tidal partial pressure of CO_2_ (PetCO_2_) observed in our study, a variable used as a noninvasive estimate of PaCO_2_ [27]. However, further research is needed, as this result could also be attributed to lactate clearance changes, the increased use of type II glycolytic fibers due to lower force rates in VK, alterations in ventilatory thresholds due to lower respiratory volumes and flows, and reduced cardiovascular capacity in women [27].

Regarding the cardiovascular system, the lower values of oxygen pulse in women suggest a diminished stroke volume and reduced peripheral vascular perfusion/extraction response to exercise [28]. These discrepancies disappeared with weight normalization, indicating a potential causal link to the likelihood of possessing a heart with smaller mass, reduced wall thickness, smaller ventricular and atrial diameters, and a lower right ventricular ejection fraction [29]. As a compensatory mechanism, the female heart attempted to increase the ejection fraction and heart rate [29]. This phenomenon was observed in our study, where women exhibited an increased heart rate in response to any absolute submaximal intensity load. Notably, this response persisted independently of weight normalization.

Finally, concerning the circulatory system, another statistically significant difference found between sexes was the total hemoglobin concentration in the quadriceps skeletal muscle (THb). That difference persisted even when normalized to the subject’s weight, revealing that men possessed a greater total Hb mass, resulting in higher blood volumes, increased cardiac output, improved oxygen diffusion, and greater oxygen differences than women [18].

These compensatory mechanisms have contributed to the absence of discernible differences in either VO_2_ or SmO_2_ between sexes, as previously observed in another study [30]. Furthermore, our results may suggest the absence of respiratory fatigue in women during a VK race, in comparison with other tests where it has been observed [31].

Consequently, cardiovascular and respiratory responses appear not to have a major impact on exercise performance in VK when comparing sexes [32], and perhaps the mitochondrial content and local muscular oxidative capacity play a contributory role [33], as each individual adapts their cardiovascular and respiratory responses to their unique physiological characteristics.

### 4.2. Performance Level Differences

The observed differences among runners based on their performance level appear to be addressed on attaining sufficient adaptations to achieve a high oxidative capacity. Top-level runners demonstrated higher values of whole-body oxygen consumption per kilogram of body (VO_2_/kg) [34]. This parameter, crucial for endurance performance, represents the amount of energy obtainable by aerobic metabolism for a given degree of fitness and oxygen availability [35,36,37] and reflects the combined capacities of the central nervous system to recruit motor units, the cardiovascular and pulmonary systems to deliver oxygen, along with the ability to extract and use that oxygen via oxidative metabolic pathways. An in-depth analysis of the VO_2_ limiting systems (cardiac output and arteriovenous oxygen difference) revealed no statistically significant differences in cardiac function (same total hemoglobin, heart rate, and oxygen pulse) among runners based on performance level in absolute terms. These results suggest that cardiovascular parameters may not be a key limiting factor in VK performance, coinciding with other submaximal exercise modalities where maximal cardiac output was not achieved, and there was no limitation in supplying oxygen to the myocardium [38]. Nevertheless, higher values in the coefficient of variation of the oxygen pulse have been observed in top-level runners, indicating superior control of their cardiovascular system, including the regulation of cardiac output and local muscle blood flow during exercise. This finding may offer a partial explanation for the increased lower limb oxygen consumption in our first quartile runners [36].

Concerning arteriovenous oxygen differences, noteworthy variations have been identified among the runners. This parameter relies on the respiratory system’s ability to supply oxygen, its transportation in blood, and its extraction and utilization in active muscles.

Our top-level runners exhibited notable pulmonary adaptations, evidenced by a higher VE, lower VD/TV, shorter TotalT, lower ET/IT, and longer IT/TotalT. These adaptations align with enhanced ventilatory efficiency, reduced breathing instability, and reduced ventilatory oscillations [39].

The lower VD/TV in top-level runners suggest that they may employ smaller increases in respiratory frequency compared to tidal volume to increase the VE in response to rising CO_2_ concentrations. Each breathing cycle is more efficient in achieving sufficient alveolar ventilation, leading to a lower flow-resistive component of ventilatory work compared to lower-level runners [23]. This improved ventilatory efficiency is reflected in lower VE/VCO_2_ ratios, indicating better ventilation/perfusion coupling during exercise [35], as well as upgraded adaptations in inspiratory musculature [40]. Top-level runners’ higher ventilatory thresholds reflects a greater reliance on oxidative metabolism, delaying the onset of fatigue due to improved hydrogen proton clearance in mitochondrial respiration [35,41].

Efficient nervous regulation of respiratory parameters is likely confirmed by shortened expiratory durations (ET) at all exercise intensities, facilitating an increased respiratory rate and minute ventilation without a proportional rise in ventilatory work by eliminating inspiratory and pharyngeal muscle actions [23]. This adjustment may explain the higher IT/TotalT ratio observed in top-level runners, primarily attributed to shorter TotalT with increasing intensities compared to low-level runners.

Concerning central inspiratory drive (TV/IT), no significant differences have been observed among the runners despite the higher acid–base imbalance in top-level runners due to increased FECO_2_ and VCO_2_. This suggests a weaker drive from peripheral and/or central chemoreceptors, making them less sensitive to hydrogen proton accumulation and allowing for greater acid–base imbalances and higher workloads [23,41].

Related to local oxygen consumption in the vastus lateralis, the top-level runners exhibited greater local oxygen consumption and higher coefficients of variation. These results could be explained by enhanced peripheral adaptations as improved capillarization per unit tissue weight, higher substrate uptake and storage, increased mitochondrial density across fiber types, greater mitochondrial crista surface, elevated oxidative enzymatic activity (3-hyroxyacyl-CoA dehydrogenase, succinate dehydrogenase, and citrate synthase), enhanced capacity for lactate production and clearance, increased neurotransmitter release and acetylcholine regulation, greater recruitment of type I motor units during submaximal exercise, higher utilization rate of oxidative metabolism over glycolytic metabolism, and better maintained nerve impulse conduction velocity despite pH changes due to H+ accumulation during high-intensity VK sections [36,41,42].

In conclusion, despite the superior pulmonary adaptations of top-level runners, the stability of partial pressure of arterial oxygen (PAO2) and the considerable potential improvement in VE suggest that the peripheral limitations may play a more significant role than central limitations in VK performance [38].

### 4.3. Vertical Kilometer Key Performance Factors

The correlation analysis conducted reveals key performance variables for both men and women collectively, given the similar performance levels in our sample. However, all correlation analyses should be approached with caution, carefully considering whether to conduct separate analyses by sex if the performance levels differ significantly. The correlation analysis with the VK final time revealed that possessing higher steady-state rates of VO_2_ and VCO_2_, coupled with higher average VE and improved nervous regulation of the respiratory cycle with shorter ET, could enable the runner to better compensate for the acid–base imbalances generated by improved oxygen and carbon dioxide transport and tolerate higher workloads. Furthermore, possessing greater local muscular adaptations facilitates greater peripheral oxygen consumption and enhanced local response to the fluctuating intensity of the VK running course. Conversely, the findings negate the implication of cardiovascular factors in the VK performance, likely attributable to the non-attainment of maximal cardiac values.

For these reasons, the causes of fatigue are likely predominantly peripheral, with multiple factors responsible for inducing this local muscular fatigue [13,38], thereby stimulating neural pathways, leading to a reduction in central motor drive and neural activation [37,43]. This theory is supported by recent investigations suggesting that VK fatigue is a combination of central and peripheral fatigue [37,44,45].

Therefore, future research should continue to focus on the analysis of afferent receptors, neural pathways, and the central integration of biomechanical, neuromuscular, and physiological factors collectively.

The sex and performance differences in VK runners have important practical applications in training. For women, it is essential to focus on improving ventilatory efficiency and the strength of respiratory muscles, given their increased respiratory workload and limitations in tidal volume. This can be achieved through specific respiratory resistance exercises. For improving runners’ performance, the development of peripheral adaptations, such as capillarization and mitochondrial density, should be prioritized to optimize local oxygen consumption. Additionally, improving the nervous regulation of breathing patterns can enhance ventilatory efficiency without increasing effort. Finally, since cardiovascular factors have a lesser impact on VK performance, training should focus on delaying peripheral muscle fatigue by enhancing aerobic endurance and oxidative capacity to improve performance at high intensities.

## 5. Conclusions

The objectives of the study were to observe the metabolic and cardiorespiratory responses during a VK field test, elucidating the differences based on performance level and sex, identifying key performance factors, and assessing the impact of fatigue. The analysis of sex differences revealed that women utilize higher heart rates to compensate for smaller pulmonary and cardiac sizes, while men employ higher cardiac outputs and saturations. Both strategies allow them to achieve an equivalent level of aerobic capacity, aerobic power, and performance. This suggests that variations in the cardiovascular and respiratory anatomies and physiologies between women and men do not seem to have a major impact on exercise performance, as each individual adapts their cardiovascular and respiratory responses to their physiological characteristics.

The differences observed among runners of varying performance levels are associated with superior aerobic capacity, better pulmonary adaptations, enhanced respiratory efficiency, improved nervous regulation of the respiratory cycle, and greater adaptations in skeletal muscle.

The correlation with the final time in the VK race in trained trail runners underscores the importance of generating respiratory and peripheral adaptations to better compensate for acid–base imbalances and tolerate higher workloads, thereby maintaining a more consistent velocity throughout the entire race.

## 6. Limitations

The main limitations of the study can be considered the low number of analyzed runners, as well as the absence of a prior maximum treadmill test before the vertical kilometer field test to determine the thresholds and percentage of maximum values at which the runners were running, providing a better understanding of the physiological demand on the athletes. Additionally, there existed a certain variability in the participants’ levels, evaluating the runners of diverse degrees of physical adaptation. While all subjects were trained athletes, none belonged to the category of professional elite trail runners.

In terms of the limitations related to NIRS usage, it is methodologically advised to measure the body fat index for the correction of the THb variable [14]; however, this has not been implemented. Additionally, NIRS was exclusively measured in the vastus lateralis despite the varied tissue oxygenation levels present in each muscle [14].

Finally, another significant limitation of the study was the lack of control over the menstrual cycle phase of the female participants, which could represent a source of variability in the results, as hormonal fluctuations may influence the physiological response to physical exercise in women.

## Figures and Tables

**Table 1:** Descriptive data of the subjects. Values are expressed as the mean ± SD.

	Men (*n* = 10)	Women (*n* = 5)
Age (years)	29 ± 2	28 ± 3
Height (cm)	174.70 ± 1.47	163.00 ± 0.95
Weight (kg)	69.50 ± 1.95	54.40 ± 1.63
BMI (kg/m^2^)	22.80 ± 0.60	20.40 ± 0.44
Training frequency (times/week)	4.66 ± 0.23	4.80 ± 0.49
Training volume (min/session)	54.40 ± 1.76	61.00 ± 8.42

SD: standard deviation; BMI: body mass index.

**Table 2:** ANCOVA test for gender differences with VK final time as the covariate. Values are expressed as the mean ± SD.

Variable	Sex	Mean ± SD	Sig	Effect Size (gANCOVA)	Statistical Power
VE peak [L/minute]	Women (*n* = 5) Men (*n* = 10)	101.70 ± 14.60 143.00 ± 12.80	<0.001	−4.04	1.00
VE mean [L/minute]	Women (*n* = 5) Men (*n* = 10)	87.20 ± 13.40 117.20 ± 13.60	<0.001	−3.31	0.97
TV peak [L]	Women (*n* = 5) Men (*n* = 10)	2.08 ± 0.21 2.94 ± 0.33	0.001	−3.91	0.98
TV mean [L]	Women (*n* = 5) Men (*n* = 10)	1.61 ± 0.18 2.32 ± 0.24	<0.001	−4.10	0.99
ET/IT mean	Women (*n* = 5) Men (*n* = 10)	1.03 ± 0.06 1.10 ± 0.06	0.034	−2.86	0.59
TV/IT peak	Women (*n* = 5) Men (*n* = 10)	3.45 ± 0.51 5.09 ± 0.49	<0.001	−4.27	1.00
TV/IT mean	Women (*n* = 5) Men (*n* = 10)	2.96 ± 0.45 4.11 ± 0.41	<0.001	−3.88	0.99
PETCO_2_ peak [mmHg]	Women (*n* = 5) Men (*n* = 10)	38.8 ± 1.30 35.8 ± 2.66	0.043	2.71	0.55
HR peak [bpm]	Women (*n* = 5) Men (*n* = 10)	187.00 ± 12.40 172.60 ± 7.38	0.012	3.45	0.78
HR mean [bpm]	Women (*n* = 5) Men (*n* = 10)	174.40 ± 8.70 165.50 ± 5.19	0.015	3.40	0.75
VO_2_/HR peak [mL/beat]	Women (*n* = 5) Men (*n* = 10)	20.30 ± 5.58 27.20 ± 5.04	0.032	−3.15	0.61
VO_2_/HR mean [mL/beat]	Women (*n* = 5) Men (*n* = 10)	15.40 ± 2.25 22.80 ± 2.54	<0.001	−3.96	0.99
THb peak [g/dL]	Women (*n* = 5) Men (*n* = 10)	12.00 ± 0.26 12.70 ± 0.41	0.010	−3.74	0.81
THb mean [g/dL]	Women (*n* = 5) Men (*n* = 10)	11.40 ± 0.76 12.50 ± 0.46	0.010	−3.41	0.81
THb CV [%]	Women (*n* = 5) Men (*n* = 10)	0.010 ± 0.005 0.006 ± 0.003	0.024	3.83	0.67

SD: standard deviation; Sig: significance level; VE: minute ventilation; TV: tidal volume; ET/IT: expiratory time-to-inspiratory time ratio; TV/IT: inspiratory flow rate; PETCO_2_: end-tidal carbon dioxide partial pressure; HR: heart rate; VO_2_/HR: oxygen pulse; THb: total hemoglobin; min: minimal value; CV: coefficient of variation. $gANCOVA={Hedge}^{\prime }s\cdot \sqrt{1-R_{ANCOVA\;adjusted}^{2}}.$

**Table 3:** Student’s *t*-test analysis was conducted with the performance level as a factor. Values are expressed as the mean ± SD.

Variable	Quartile	Mean ± SD	Sig	Effect Size (Hedges’ g)	Statistical Power
VK final time [minutes]	Q1 (*n* = 5) Q2–Q4 (*n* = 10)	46.08 ± 1.71 58.92 ± 5.91	<0.001	−2.41	1.00
Velocity min [m/s]	Q1 (*n* = 5) Q2–Q4 (*n* = 10)	1.80 ± 1.22 1.32 ± 0.37	0.009	1.41	0.18
Velocity mean [m/s]	Q1 (*n* = 5) Q2–Q4 (*n* = 10)	5.91 ± 0.40 4.77 ± 0.47	<0.001	2.36	0.99
VO_2_/kg peak [mL/kg/minute]	Q1 (*n* = 5) Q2–Q4 (*n* = 10)	71.50 ± 3.23 58.70 ± 3.97	<0.001	3.21	1.00
VO_2_/kg mean [mL/kg/minute]	Q1 (*n* = 5) Q2–Q4 (*n* = 10)	62.50 ± 4.31 50.70 ± 2.62	<0.001	3.35	0.99
VCO_2_/kg peak [mL/kg/minute]	Q1 (*n* = 5) Q2–Q4 (*n* = 10)	66.70 ± 7.73 53.00 ± 2.75	<0.001	2.58	0.84
VCO_2_/kg mean [mL/kg/minute]	Q1 (*n* = 5) Q2–Q4 (*n* = 10)	51.90 ± 3.91 40.90 ± 3.21	<0.001	2.98	0.99
VE peak [L/minute]	Q1 (*n* = 5) Q2–Q4 (*n* = 10)	142.70 ± 15.30 120.30 ± 25.30	0.05	0.93	0.46
VE mean [L/minute]	Q1 (*n* = 5) Q2–Q4 (*n* = 10)	119.10 ± 14.50 99.50 ± 19.50	0.038	1.01	0.51
VD/TV mean	Q1 (*n* = 5) Q2–Q4 (*n* = 10)	0.23 ± 0.01 0.26 ± 0.03	0.045	−0.96	0.75
TotalT min [seconds]	Q1 (*n* = 5) Q2–Q4 (*n* = 10)	0.79 ± 0.23 0.96 ± 0.11	0.038	−1.01	0.25
TotalT mean [seconds]	Q1 (*n* = 5) Q2–Q4 (*n* = 10)	0.99 ± 0.31 1.19 ± 0.11	0.049	−0.94	0.20
ET/IT peak	Q1 (*n* = 5) Q2–Q4 (*n* = 10)	1.24 ± 0.10 1.43 ± 0.11	0.004	−1.64	0.84
IT/TotalT min	Q1 (*n* = 5) Q2–Q4 (*n* = 10)	0.45 ± 0.02 0.42 ± 0.02	0.025	1.14	0.72
FECO_2_ peak [%]	Q1 (*n* = 5) Q2–Q4 (*n* = 10)	4.73 ± 0.40 4.32 ± 0.27	0.021	1.19	0.41
FECO_2_ mean [%]	Q1 (*n* = 5) Q2–Q4 (*n* = 10)	3.81 ± 0.32 3.51 ± 0.25	0.037	1.02	0.35
SmO_2_ min [%]	Q1 (*n* = 5) Q2–Q4 (*n* = 10)	9.62 ± 3.37 24.70 ± 13.10	0.003	−1.23	0.87
SmO_2_ mean [%]	Q1 (*n* = 5) Q2–Q4 (*n* = 10)	28.30 ± 5.38 46.20 ± 15.4	0.004	−1.23	0.86
VE/VCO_2_ mean	Q1 (*n* = 5) Q2–Q4 (*n* = 10)	34.40 ± 3.05 37.70 ± 2.72	0.028	−1.10	0.43
VO_2_/HR CV [%]	Q1 (*n* = 5) Q2–Q4 (*n* = 10)	16.30 ± 1.71 12.10 ± 4.62	0.027	0.93	0.23
SmO_2_ CV [%]	Q1 (*n* = 5) Q2–Q4 (*n* = 10)	41.20 ± 10.20 19.20 ± 12.50	0.005	1.71	0.74

SD: standard deviation; Sig: significance level; VK: vertical kilometer; VO_2_: oxygen uptake; VCO_2_: carbon dioxide production; VE: minute ventilation; VD/TV: dead space-to-tidal volume ratio; TotalT: total time of respiratory cycle; ET/IT: expiratory time-to-inspiratory time ratio; IT/TotalT: inspiratory duty cycle; FECO_2_: expiratory fractions of carbon dioxide; SmO_2_: muscle oxygen saturation index. VE/VCO_2_: minute ventilation and carbon dioxide production ratio; VO_2_/HR: oxygen pulse; CV: coefficient of variation; Q1: first quartile; Q2–Q4: remaining quartiles; min: minimal value.

## Data Availability

The datasets generated during and/or analyzed during the current study are available from the corresponding author on reasonable request.
